# Supplementary material for: HIF-2α-pVHL complex reveals broad genotype-phenotype correlations in HIF-2α-driven disease
Source: Nat Commun. 2018 Aug 22;9:3359. doi: 10.1038/s41467-018-05554-1 (PMC6105673; doi:10.1038/s41467-018-05554-1)
Supplement: Supplementary file 1 — Supplementary Information [file 41467_2018_5554_MOESM1_ESM.pdf]

**HIF-2α-pVHL complex reveals broad genotype-phenotype correlations in HIF-2α-driven disease**

Tarade et al.

**Supplementary Information**

## Supplementary Figures

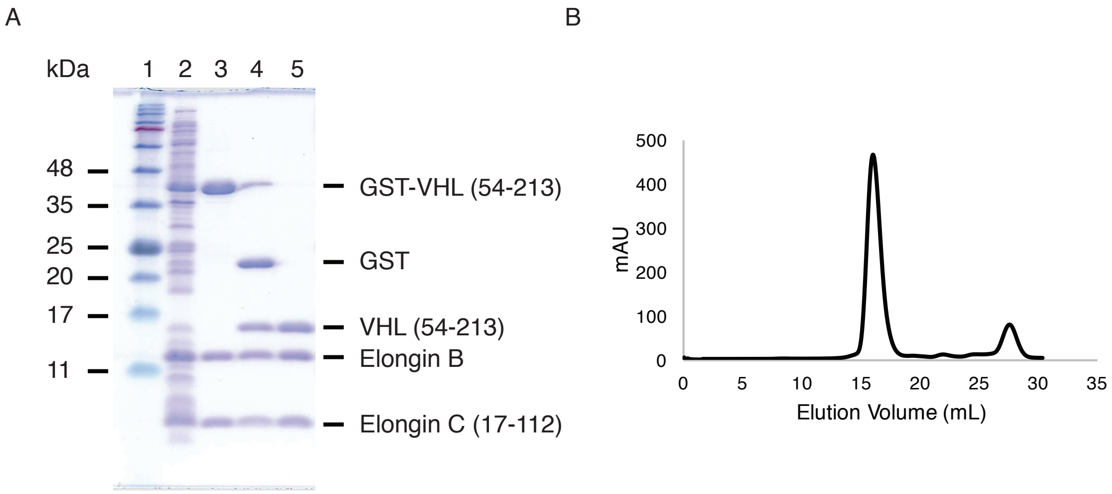


**Supplementary Figure 1. Purification of pVHL-elongin B-elongin C (VBC) complex.** GST-tagged $pVHL_{19}$ (residues 54-213) was co-expressed with untagged elongin B and elongin C (17-112) in BL21(DE3) *E. coli*. (A) Glutathione sepharose resin was used to affinity purify the GST-VBC complex. The GST-tag was cleaved from pVHL via incubation with thrombin. GST was removed from the protein solution via affinity purification. Successful cleavage of the GST-tag and purity of the sample was determined by SDS-PAGE analysis followed by Coomassie staining. Lane 1 = protein ladder; lane 2 = flow-through during affinity purification with glutathione sepharose resin; lane 3 = GST-VBC complex eluted from glutathione sepharose resin; lane 4 = VBC complex following incubation with 1 U/mg thrombin for 60 hours at 4 °C; lane 5 = VBC complex following affinity purification to remove GST. (B) Complex was purified to homogeneity via size exclusion chromatography. VBC complex had an elution volume of 16 mL. mAU = milli absorption units.

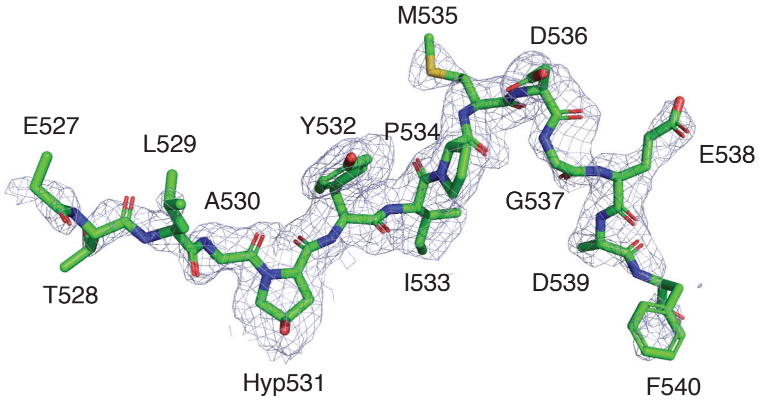


**Supplementary Figure 2. Electron density of HIF-2α peptide.** The peptide backbone is displayed as a stick model. The orientation of the peptide is identical to that shown in Fig. 3b. Carbon is shown in green, nitrogen is shown in blue, oxygen is shown in red, and sulfur is shown in yellow. The composite omit (2Fo – Fc) electron density map is contoured at 1.0 σ and shown in blue mesh. The 2Fo – Fc electron density map is generated with 5% of atoms omitted.

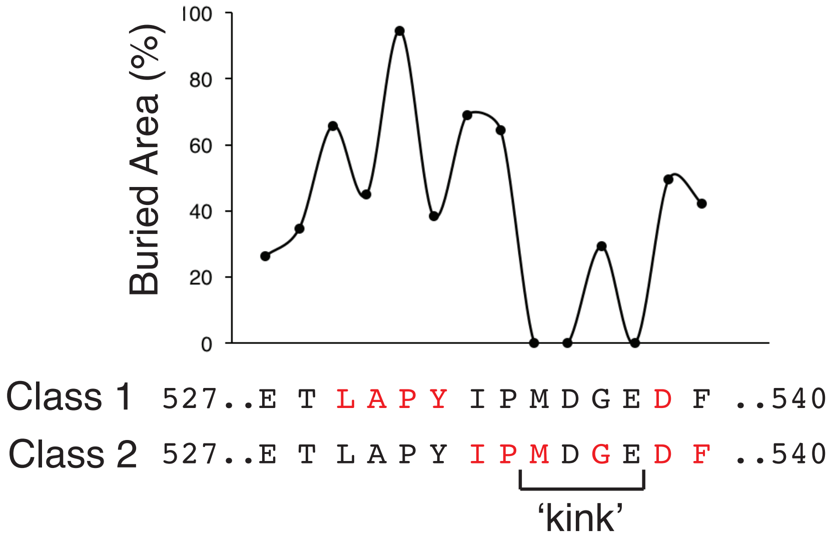


**Supplementary Figure 3. Analysis of the HIF-2α-pVHL interface reveals a kink region.** The interface between HIF-2αOH peptide and VBC complex was analyzed via Protein Interfaces, Surfaces and Assemblies (PISA). Buried area percentage is calculated as a ratio of buried surface area (Å²) over accessible surface area (Å²).

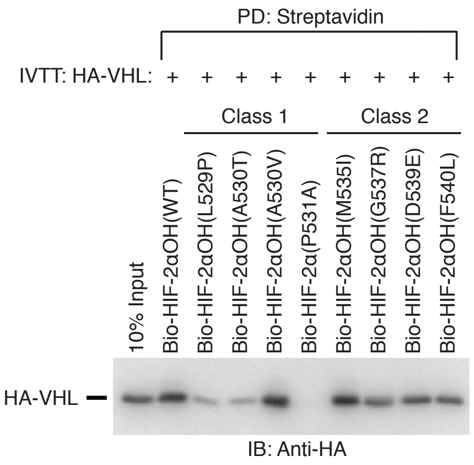


**Supplementary Figure 4. Affinity of HIF-2α mutants for pVHL is correlated with disease phenotype.** Biotinylated HIF-2αOH peptides (523-541) were immobilized on streptavidin- agarose beads and incubated with *in vitro* transcribed and translated (IVTT) pVHL. Streptavidin beads were pulled down (PD) and levels of HA-tagged pVHL were visualized via immmunoblotting (IB). Binding of pVHL occurred in 0.02% (v/v) Tween-20 buffer and beads were washed with 0.1% (v/v) Tween-20 buffer. The presented western blot is representative of three experiments.

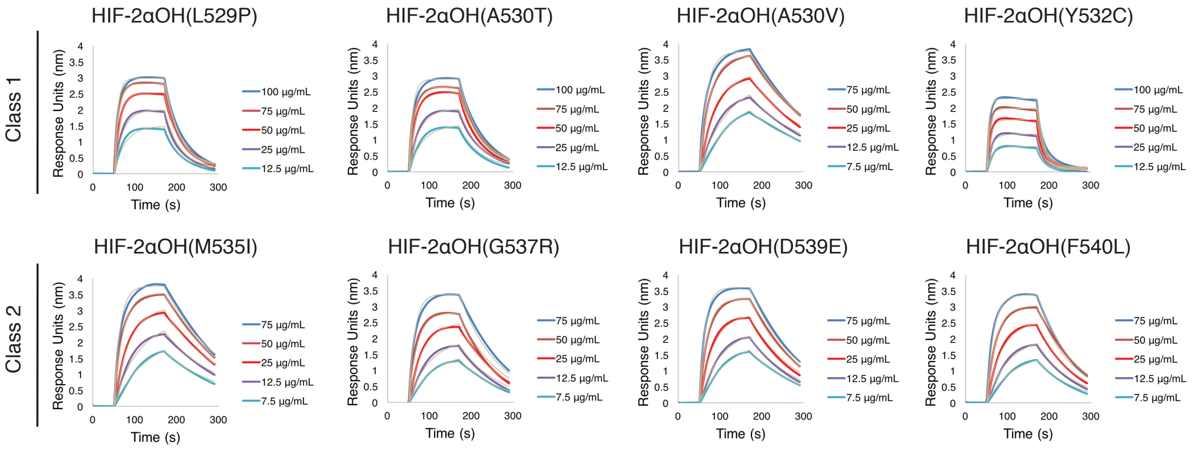


**Supplementary Figure 5. Class 1 mutations more adversely disrupt HIF-2α peptide binding to pVHL than class 2 mutations.** Biolayer interferometry kinetic analysis of pVHL-elongin B-elongin C (VBC) complex binding to biotinylated HIF-2αOH peptides (523-541). Biotinylated peptides were coupled to streptavidin- coated biosensors and monitored for binding to VBC complex at the indicated concentrations. The data were analyzed based on a 1:1 binding model using the BLItz Pro software with the fitted curves shown as gray lines. Sensorgrams are representative of three experiments conducted with independently purified proteins.

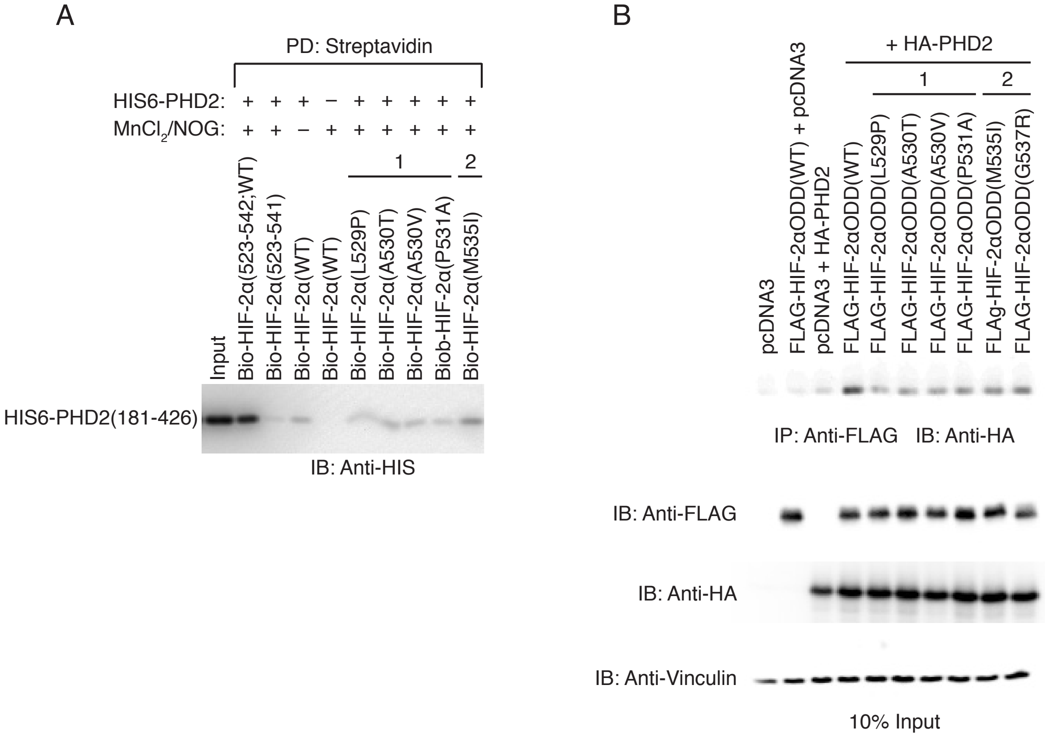


**Supplementary Figure 6. HIF-2α mutations disrupt PHD2 binding.** (A) Biotinylated HIF-2α peptides (523-542, except where noted) were immobilized on streptavidin- agarose beads and incubated with purified $HIS_6$-PHD2 (181-426) in the presence or absence of 1 mM $MnCl_2$ and 1 mM *N*-oxalylglycine (NOG). Streptavidin beads were pulled down (PD) and levels of $HIS_6$-tagged PHD2 were visualized via immmunoblotting (IB). The presented western blot is representative of three experiments. (B) HEK293a cells were co-transfected with HA-PHd2 and FLAG-HIF-2α ODD constructs. Flag was immunoprecipitated (IP) and levels of HA-tagged PHD2 were visualized via immunoblotting (IB). The presented western blot is representative of three experiments.

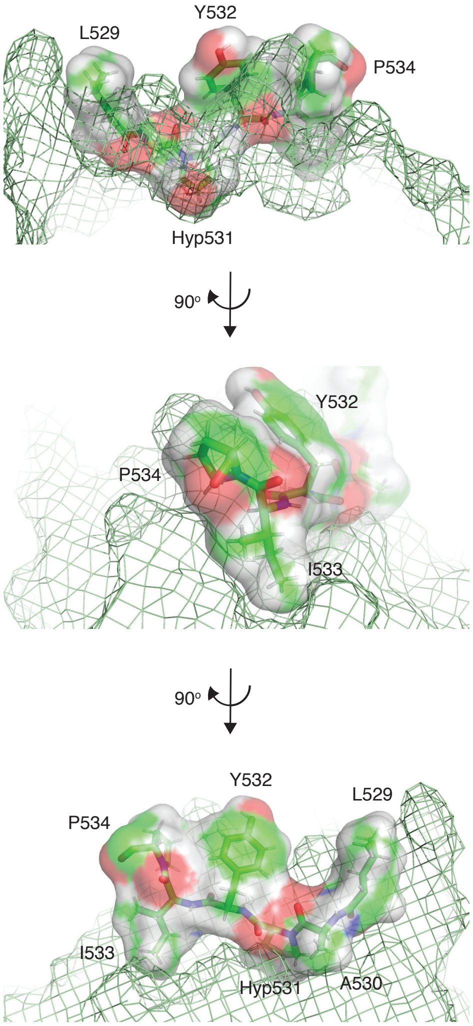


**Supplementary Figure 7. I533 and P534 make significant contacts with pVHL.** The HIF-2α peptide is shown as a stick model superimposed with a surface representation. Carbon, oxygen and nitrogen atoms are shown in green, red, and blue, respectively. The pVHL complex is displayed as a surface mesh.

## Figure 4b

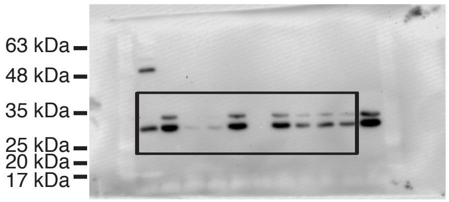


Anti-HA

## Figure 5a

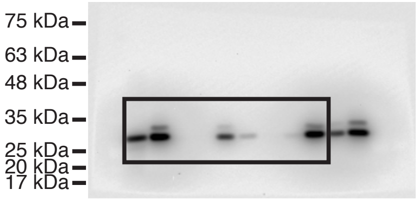


Anti-HA

## Figure 5b

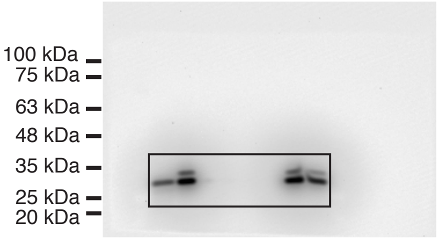


Anti-HA

## Figure 5c

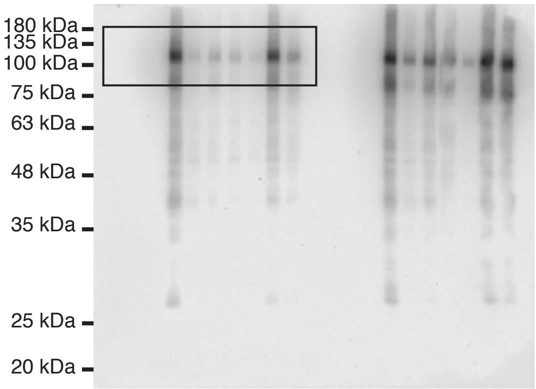


Anti-HA

## Figure 5c

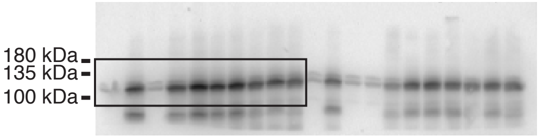


Anti-HA

## Figure 5c

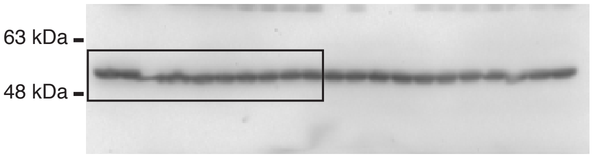


Anti-Tubulin

## Figure 5c

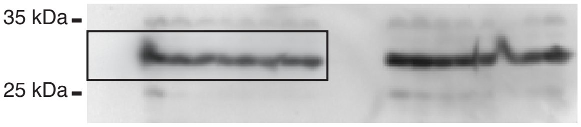


Anti-FLAG

## Supplementary Figure 4

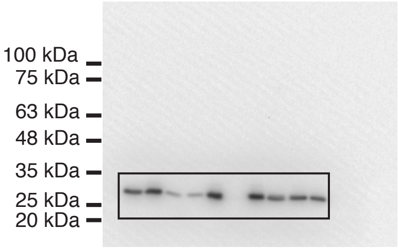


Anti-HA

## Supplementary Figure 6a

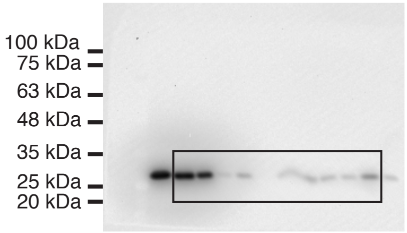


Anti-HIS

## Supplementary Figure 6B

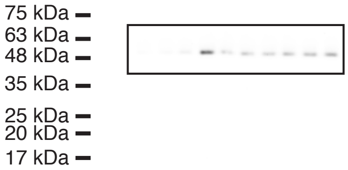


IP:Anti-FLAG
IB: Anti-HA

## Supplementary Figure 6B

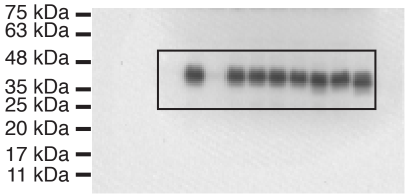


Anti-FLAG

## Supplementary Figure 6B

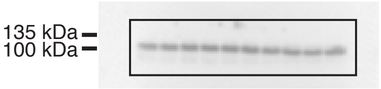


Anti-Vinculin

## Supplementary Figure 6B

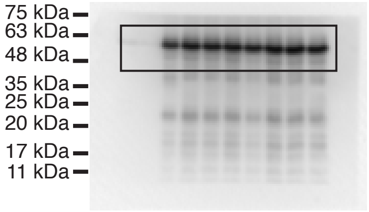


Anti-HA

**Supplementary Figure 8. Uncropped western blots.**

**Supplementary Table 1. Mutation prediction software analysis of HIF-2α mutants.**

| | Nucleotide Sequence Variant | Amino Acid Sequence Variant | SIFT Prediction (score) Threshold = 0.05 | MutationTaster Prediction (probability value) | PROVEAN (score) Threshold = -4.1 | PolyPhen-2 Prediction (score) |
|---|---|---|---|---|---|---|
| **Class 1** | c.1588G>A | A530T | Damaging (0.000) | Disease Causing (1) | Neutral (-3.43) | Probably Damaging (1.00) |
| | c.1595A>G | Y532C | Damaging (0.000) | Disease Causing (1) | Deleterious (-7.83) | Probably Damaging (1.00) |
| | c.1625T>C | L542P | Damaging (0.000) | Disease Causing (1) | Deleterious (-5.77) | Probably Damaging (1.00) |
| | c.1615G>A | D539N | Damaging (0.002) | Disease Causing (1) | Deleterious (-4.25) | Probably Damaging (1.00) |
| | c.1586T>C | L529P | Damaging (0.000) | Disease Causing (1) | Deleterious (-5.75) | Probably Damaging (1.00) |
| | c.1589C>T | A530V | Damaging (0.000) | Disease Causing (1) | Neutral (-3.36) | Probably Damaging (1.00) |
| | c.1591C>T | P531S | Damaging (0.000) | Disease Causing (1) | Deleterious (-6.89) | Probably Damaging (1.00) |
| | c.1592C>T | P531L | Damaging (0.000) | Disease Causing (1) | Deleterious (-8.64) | Probably Damaging (1.00) |
| | c.1589C>A | A530E | Damaging (0.000) | Disease Causing (1) | Deleterious (-4.30) | Probably Damaging (1.00) |
| | c.1121T>A | F374Y | Tolerated (0.135) | Disease Causing (0.999) | Neutral (-1.02) | Benign (0.074) |
| | c.1615G>T | D539Y | Damaging (0.000) | Disease Causing (1) | Deleterious (-6.77) | Probably Damaging (1.00) |
| | c.1592C>G | P531R | Damaging (0.000) | Disease Causing (1) | Deleterious (-7.80) | Probably Damaging (1.00) |

| | | | | | | |
|---|---|---|---|---|---|---|
| | c.1591C>G | P531A | Damaging (0.000) | Disease Causing (1) | Deleterious (-6.99) | Probably Damaging (1.00) |
| | c.212C>A | S71Y | Damaging (0.005) | Disease Causing (1) | Neutral (-3.15) | Probably Damaging (0.994) |
| | c.1591C>A | P531T | Damaging (0.000) | Disease Causing (1) | Deleterious (-6.93) | Probably Damaging (1.00) |
| | c.1104G>A | M368I | Tolerated (0.232) | Disease Causing (0.657) | Neutral (-0.75) | Benign (0.022) |
| | c.1556C>T | T519M | Tolerated (0.113) | Disease Causing (0.843) | Neutral (-1.32) | Benign (0.157) |
| | c.1630C>T | P544S | Tolerated (0.479) | Disease Causing (1) | Neutral (-1.40) | Probably Damaging (1.00) |
| **Class 2** | c.1609G>T | G537W | Damaging (0.000) | Disease Causing (1) | Neutral (-2.79) | Probably Damaging (1.00) |
| | c.1604T>C | M535T | Damaging (0.000) | Disease Causing (1) | Deleterious (-5.25) | Probably Damaging (1.00) |
| | c.1620C>G | F540L | Damaging (0.003) | Disease Causing (1) | Deleterious (-5.26) | Probably Damaging (1.00) |
| | c.1603A>G | M535V | Damaging (0.000) | Disease Causing (1) | Neutral (-3.54) | Probably Damaging (0.999) |
| | c.1609G>A | G537R | Damaging (0.000) | Disease Causing (1) | Neutral (-2.76) | Probably Damaging (1.00) |
| | c.1605G>A | M535I | Damaging (0.000) | Disease Causing (1) | Neutral (-3.54) | Probably Damaging (0.999) |
| | c.1617C>G | D539E | Damaging (0.001) | Disease Causing (1) | Neutral (-3.44) | Probably Damaging (1.00) |
| | C.1597A>G | I533V | Damaging (0.002) | Disease Causing (1) | Neutral (-0.87) | Probably Damaging (1.00) |
| | | P534L | Damaging (0.001) | Disease Causing (1) | Deleterious (-7.44) | Probably Damaging (1.00) |

**Supplementary Table 2. Data collection and refinement statistics.**

| | VHL-EloB-EloC-HIF2α |
|---|---|
| **Data collection** | |
| Space group | $P4_32_12$ |
| Cell dimensions | |
| *a*, *b*, *c* (Å) | 59.8, 59.8, 246.1 |
| α, β, γ (°) | 90, 90, 90 |
| Resolution (Å)[a] | 42.9-2.0 (2.05-2.00) |
| $R_{meas}$ (%)[a,b] | 9.5 (91.2) |
| $I / \sigma(I)$[a] | 13.2 (1.3) |
| $CC_{1/2}$[a] | 99.7 (70.1) |
| Completeness (%)[a] | 99.3 (92.2) |
| Total no. of reflections[a] | 265,217 (7950) |
| No. of unique reflections[a] | 31,053 (2084) |
| Redundancy[a] | 8.5 (3.8) |
| **Refinement** | |
| Resolution (Å) | 42.9-2.0 |
| $R_{work}$ / $R_{free}$ (%) | 19.9/23.6 |
| No. atoms (non-hydrogen) | 2895 |
| Protein | 2793 |
| Water | 102 |
| *B*-factor (Å$^2$) | 46.7 |
| Protein | 47.0 |
| Water | 39.7 |
| R.m.s. deviation | |
| Bond lengths (Å) | 0.012 |
| Bond angles (°) | 1.10 |
| Ramachandran Plot | |
| Most favored region (%) | 96.5 |
| Additional allowed region (%) | 3.5 |
| Outliers (%) | 0.0 |
| Rotamer outliers (%) | 1.7 |
| Clashscore | 1.0 |
| Molprobity score | 1.4 |

[a]Values in parentheses are for highest-resolution shell (2.05-2.00)

[b]$R_{meas}$: multiplicity-independent R = Sum(Sqrt(N/N-1))(|Ihl - <Ih>|))/Sum(<Ih>)

**Supplementary Table 3. List of Primers.**

| Primer Name | Primer Sequence (5'-3') |
|---|---|
| HIF2α G537R FWD | CTATATCCCCATGGACAGGGAAGACTTCCAGC |
| HIF2α G537R REV | GCTGGAAGTCTTCCCTGTCCATGGGGATATAG |
| HIF2α M535I FWD | CACCCTATATCCCCATAGACGGGGAAGACTTC |
| HIF2α M535I REV | GAAGTCTTCCCCGTCTATGGGGATATAGGGTG |
| HIF2α A530V FWD | GACTTGGAGACACTGGTACCCTATATCCCCATG |
| HIF2α A5350V REV | CATGGGGATATAGGGTACCAGTGTCTCCAAGTC |
| HIF2α A530T FWD | GGACTTGGAGACACTGACACCCTATATCCCCATG |
| HIF2α A530T REV | CATGGGGATATAGGGTGTCAGTGTCTCCAAGTCC |
| HIF2α L529P FWD | GGACTTGGAGACACCGGCACCCTATATCC |
| HIF2α L529P REV | GGATATAGGGTGCCGGTGTCTCCAAGTCC |
| HIF2α (390-555) FWD | GGTGGTGGATCCTTCACCAAGCTAAAGGAGGAGC |
| HIF2α (390-555) REV | ATTATTGAATTCCTACTCCGCCAAGAGCCGCT |
