# Supplementary material for: HIF-2α-pVHL complex reveals broad genotype-phenotype correlations in HIF-2α-driven disease
Source: Nat Commun. 2018 Aug 22;9:3359. doi: 10.1038/s41467-018-05554-1 (PMC6105673; doi:10.1038/s41467-018-05554-1)
Supplement: Supplementary file 3 — Description of Additional Supplementary Files [file 41467_2018_5554_MOESM3_ESM.pdf]

Description of Additional Supplementary Files

File Name: Supplementary Data 1
Description: Detailed clinical information of patients with HIF-2α mutations
